# 1 Patient With Single Coronary Artery, Giant Coronary Artery Aneurysm, Contained Rupture, and Fistula

**DOI:** 10.1016/j.jaccas.2024.102396

**Published:** 2024-06-05

**Authors:** Nikita Mishra, Yasmin Hamirani, Partho P. Sengupta, Leonard Y. Lee, Sabahat Bokhari

**Affiliations:** a Division of Cardiovascular Diseases and Hypertension, Rutgers University – Robert Wood Johnson Medical School, New Brunswick, New Jersey, USA; b Department of Surgery, Rutgers University–Robert Wood Johnson Medical School, New Brunswick, New Jersey, USA

**Keywords:** coronary artery aneurysm, coronary artery fistula, coronary CTA, giant coronary artery, single coronary artery

## Abstract

Single coronary artery, giant coronary artery aneurysm, and coronary cameral fistula are rare congenital anomalies, and can cause a range of presentations. To our knowledge, this is the first reported case of all 3 entities occurring simultaneously in 1 patient, with largely unknown implications. Multimodal imaging was essential in prompt diagnosis and management.

## Graphical abstract

**Figure:**
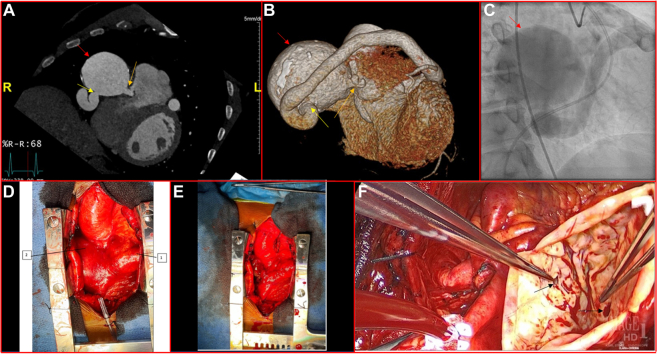

A 56-year-old man presented to the emergency department after a chest x-ray performed at an urgent care for epigastric pain was concerning for a “mass around the heart.” The patient’s vital signs and laboratory results were within normal limits, including a normal high-sensitivity troponin of 7 nanograms/liter (ng/L). His EKG demonstrated normal sinus rhythm, and computed tomography of the chest suggested a possible fistula between the left main and right coronary arteries. Coronary computed tomography angiography allowed for improved visualization of an aneurysmal single coronary artery (SCA) originating from the left coronary cusp of the aorta (yellow arrow in Figures 1A and 1B), coursing through the right atrioventricular groove and terminating in a fistula with the right ventricle (orange arrow in Figures 1A and 1B), with a ruptured segment contained within the myocardium measuring 6.5 × 6.2 cm (red arrow in Figures 1A and 1B). The patient underwent left heart catheterization, which redemonstrated the aneurysmal SCA and contained rupture (red arrow in Figure 1C), and confirmed the absence of coronary artery disease. Right heart catheterization was also performed and showed a significant oxygen step-up from 68.75% in the superior vena cava to 80.16% in the main pulmonary artery, confirming the presence of a left-to-right shunt caused by the SCA. Finally, transthoracic echocardiogram demonstrated both systolic and diastolic flow within the vessel, further suggestive of shunt physiology.

**Figure 1:**
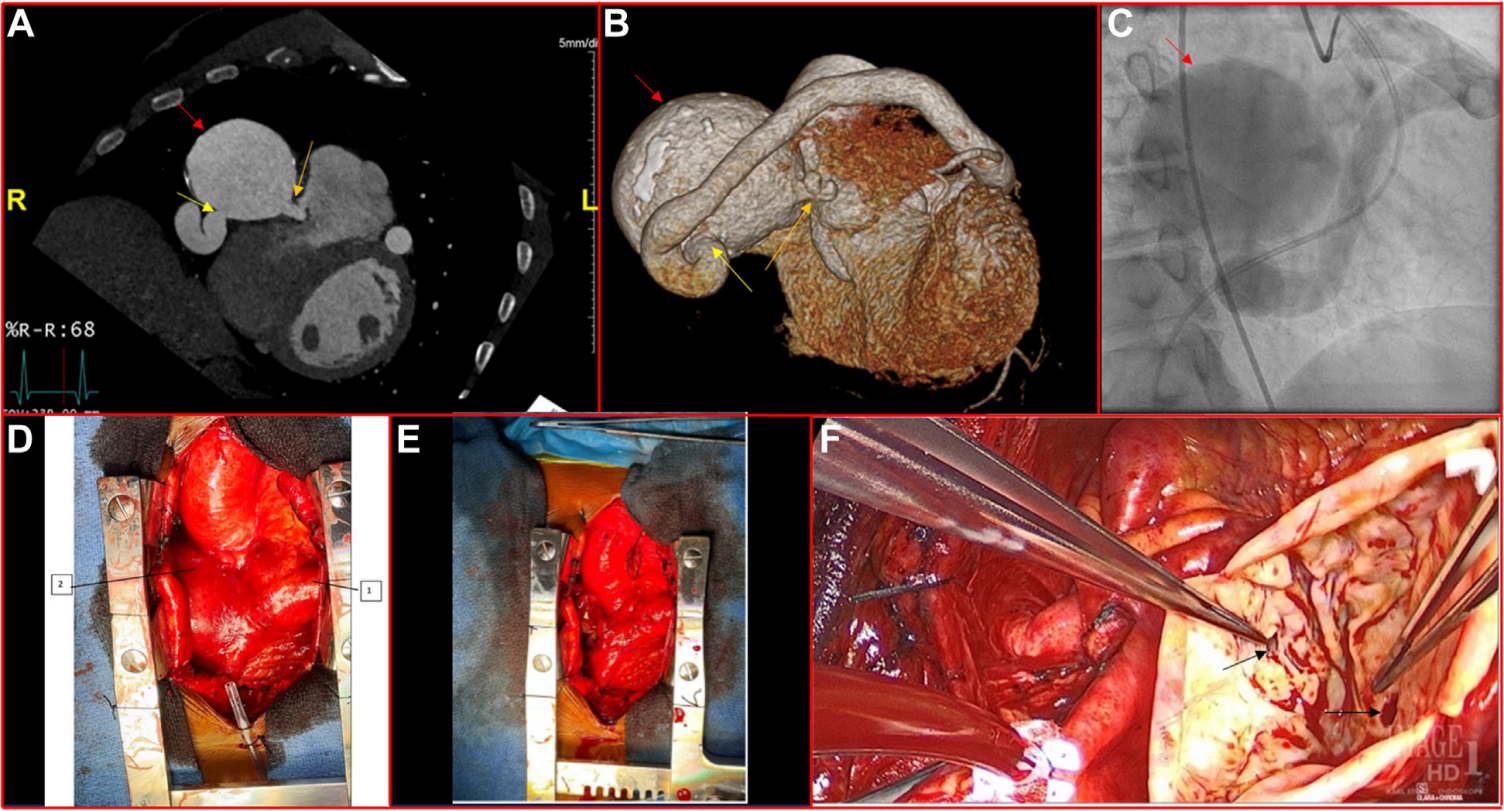
Coronary CTA, Cardiac Catheterization, and Intra-operative Images (A) Computed tomography angiography of the heart and coronary arteries demonstrating the contained rupture (red arrow) with connection to the right ventricle (orange arrow) and connection to the aneurysmal single coronary artery (yellow arrow). (B) Three-dimensional reconstruction of coronary computed tomography angiography redemonstrating contained rupture and connections to the right ventricle and aneurysmal single coronary artery. (C) Left heart catheterization image demonstrating aneurysmal single coronary artery and contained rupture. (D) Intraoperative image demonstrating aneurysmal single coronary artery (1) feeding into contained rupture (2). (E) Intraoperative image after arteriotomy and ligation. (F) Opened contained rupture with connections to right ventricle and coronary artery indicated by forceps and solid arrows.

SCA, coronary artery aneurysm, and fistula are all rare congenitally acquired phenomena that are each associated with an increased risk of adverse cardiac events, and combined with the presence of contained rupture, this case is especially both unique and worrisome. SCA predisposes to sudden cardiac death if the aberrant coronary artery courses between outflow tracts, has an acute takeoff angle, has a slit-like orifice, or follows an intramural course.^1^ Coronary artery aneurysm has been cited in 0.3% to 4.9% of all patients undergoing coronary angiography, and more than 90% of these in adults are attributed to atherosclerosis.^2^ In addition, the diameter of the aneurysmal artery was 14 mm, thus defining it as “giant” by exceeding 8 mm. Coronary artery aneurysms have independently been associated with obstructive coronary artery disease, myocardial infarction, arrhythmias, and sudden cardiac death.^2^ Given the novelty of this case, decisions regarding management required multidisciplinary discussions between cardiology and cardiothoracic surgery.

Treatment for SCA depends on the patient’s symptom severity. In this case, surgical management was pursued given the patient’s symptoms and complex anatomy predisposing him to sudden cardiac death. During surgery, the giant aneurysmal coronary artery was visualized coursing inferiorly across the right ventricular outflow tract (denoted by the 1 arrow in Figure 1D) and feeding into the contained rupture (denoted by the 2 arrow in Figure 1D). Arteriotomy and ligation were performed, with the results shown in Figure 1E. This was accomplished by opening the contained rupture, with connections to the right ventricle and aneurysmal artery shown in Figure 1F (denoted by the forceps and solid arrows). The wall of the contained rupture was resected and sent off as a specimen. Pathology was notable for hypertrophied myocardium with fibrosis and focal calcification, and fragments of calcified fibroconnective tissue. The patient followed up at general cardiology clinic and was noted to have preserved left ventricular function on postoperative transthoracic echocardiogram.

## Funding Support and Author Disclosures

The authors have reported that they have no relationships relevant to the contents of this paper to disclose.
